# Pruritus of the Vulva

**Published:** 1843-11

**Authors:** 


*Pruritus of the Vulva.*—This is a not unfrequent and a most distressing complaint. In the treatment of it, Lisfranc places his chief reliance on an occasional bleeding from the arm. Lotions of alum, or of nitrate of silver, are sometimes very useful; but on the whole, Raspail's remedy—five parts of starch and one of camphor—is still better as a local application. It may be applied two or three times a day; the proportion of the camphor should be made to vary, according to the irritable state of the affected parts. Fumigations with the vapour of sulphur will sometimes effect a cure, when all other remedies fail. Let it ever be remembered that pruritus of the vulva is not unfrequently connected with some morbid affection of the uterus, and therefore that our chief attention must be directed to the removal of the cause. An opiate enema is sometimes a valuable remedy. Lisfranc alludes to a very obstinate case, where a very rapid cure was obtained from the administration of sulphate of quinine, after other means had quite failed.—*Amer. Journ. of the Med. Sci.*, from *Med. Chirurg. Rev.*, April, 1843, from Lisfranc's *Clinique Chirurg.*, tom. ii.

